# Assessing the mechanism of citywide test-trace-isolate Zero-COVID policy and exit strategy of COVID-19 pandemic

**DOI:** 10.1186/s40249-022-01030-7

**Published:** 2022-10-04

**Authors:** Pei Yuan, Yi Tan, Liu Yang, Elena Aruffo, Nicholas H. Ogden, Guojing Yang, Haixia Lu, Zhigui Lin, Weichuan Lin, Wenjun Ma, Meng Fan, Kaifa Wang, Jianhe Shen, Tianmu Chen, Huaiping Zhu

**Affiliations:** 1 Laboratory of Mathematical Parallel Systems (LAMPS), Department of Mathematics and Statistics, York University, 4700 Keele Street, Toronto, ON M3J1P3 Canada; 2 Canadian Centre for Diseases Modeling (CCDM), York University, Toronto, Canada; 3 School of Mathematics and Statistics, Northeast Normal University, Changchun, Jilin China; 4 Public Health Risk Sciences Division, National Microbiology Laboratory, Public Health Agency of Canada, Saint-Hyacinthe, Canada; 5 Key Laboratory of Tropical Translational Medicine of Ministry of Education and School of Tropical Medicine and Laboratory Medicine, The First Affiliated Hospital of Hainan Medical University, Hainan Medical University, Haikou, Hainan China; 6 School of Arts and Science, Suqian University, Suqian, Jiangsu China; 7 School of Mathematical Science, Yangzhou University, Yangzhou, Jiangsu China; 8 School of Mathematics and Statistics, Fujian Normal University, Fuzhou, Fujian China; 9 Department of Public Health and Preventive Medicine, School of Medicine, Jinan University, Guangzhou, Guangdong China; 10 Disease Control and Prevention Institute, Jinan University, Guangzhou, Guangdong China; 11 School of Mathematics and Statistics, Southwest University, Chongqing, China; 12 School of Public Health and State Key Laboratory of Molecular Vaccinology and Molecular Diagnostics, Xiamen University, Xiamen, Fujian China

**Keywords:** COVID-19, Transmission model, Zero-COVID policy, Citywide testing, Test-trace-isolate, Exit strategy

## Abstract

**Background:**

Countries that aimed for eliminating the cases of COVID-19 with test-trace-isolate policy are found to have lower infections, deaths, and better economic performance, compared with those that opted for other mitigation strategies. However, the continuous evolution of new strains has raised the question of whether COVID-19 eradication is still possible given the limited public health response capacity and fatigue of the epidemic. We aim to investigate the mechanism of the Zero-COVID policy on outbreak containment, and to explore the possibility of eradication of Omicron transmission using the citywide test-trace-isolate (CTTI) strategy.

**Methods:**

We develop a compartmental model incorporating the CTTI Zero-COVID policy to understand how it contributes to the SARS-CoV-2 elimination. We employ our model to mimic the Delta outbreak in Fujian Province, China, from September 10 to October 9, 2021, and the Omicron outbreak in Jilin Province, China for the period from March 1 to April 1, 2022. Projections and sensitivity analyses were conducted using dynamical system and Latin Hypercube Sampling/ Partial Rank Correlation Coefficient (PRCC).

**Results:**

Calibration results of the model estimate the Fujian Delta outbreak can end in 30 (95% confidence interval *CI*: 28–33) days, after 10 (95% *CI*: 9–11) rounds of citywide testing. The emerging Jilin Omicron outbreak may achieve zero COVID cases in 50 (95% *CI*: 41–57) days if supported with sufficient public health resources and population compliance, which shows the effectiveness of the CTTI Zero-COVID policy.

**Conclusions:**

The CTTI policy shows the capacity for the eradication of the Delta outbreaks and also the Omicron outbreaks. Nonetheless, the implementation of radical CTTI is challenging, which requires routine monitoring for early detection, adequate testing capacity, efficient contact tracing, and high isolation compliance, which constrain its benefits in regions with limited resources. Moreover, these challenges become even more acute in the face of more contagious variants with a high proportion of asymptomatic cases. Hence, in regions where CTTI is not possible, personal protection, public health control measures, and vaccination are indispensable for mitigating and exiting the COVID-19 pandemic.

**Graphical Abstract:**

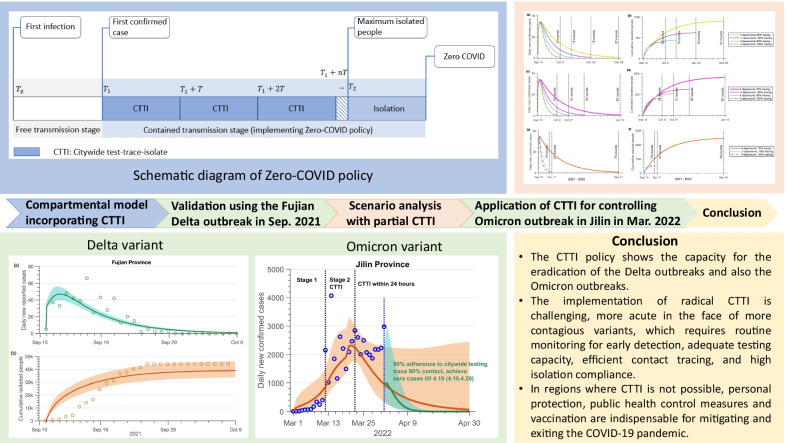

**Supplementary Information:**

The online version contains supplementary material available at 10.1186/s40249-022-01030-7.

## Background

Since the first COVID-19 case was reported in 2019, new variants have been found in different parts of the world. Many of them were classified as Variants of Concern (VOC) due to the higher transmissibility and lower response to vaccination and public health measures [1]. The lifting of non-pharmaceutical interventions (NPIs), new emerging variants, and waning and lower efficacy of vaccination lead to the broad resurgence of infections [2, 3]. Some countries, like China, Australia, Japan, and South Korea, adopted an elimination strategy at the early stage of pandemic, the so-called “Zero-COVID strategy”, aimed to eradicate the virus as soon as new cases were detected, while many others opted for mitigation measures to minimize the number of cases [4–7].

The Zero-COVID strategy includes measures such as mass testing, detailed contact tracing using the most advanced technologies, and strict control measures upon detection of new cases, and this strategy appears to be more beneficial than less stringent measures [4, 7, 8]. Oliu-Barton et al. (2021) found that the countries that opted for the elimination strategy had 25 times lower COVID-19 death rates, compared to that with the mitigation strategy [7]. The report from Institut économique Molinari highlights that the number of deaths under the Zero-COVID strategy is 44 times lower than the other strategies, and the tough control measures do not affect economic growth [8]. On the other hand, countries with weaker NPIs of mild testing and tracing processes reported multiple waves despite the high vaccination coverage.

There have been modeling studies [9–11] that concluded that continuous testing, rigorous contact tracing, and prompt quarantine are crucial to containing the spread of the infection. However, it was argued that contact tracing is an imperfect tool for controlling COVID-19 transmission since it relies on population adherence. Davis et al. [9] found that there are no benefits in increasing coverage of traced contacts or speeding up testing with poor reporting rate and adherence rate to isolation. Also, symptom-driven testing is doubted due to the pre- and asymptomatic transmission [10], accounting for 59% of community infections [12]. Weekly population-wide testing is found to be efficient to contain the transmission within a few weeks when combined with contact tracing and isolation [13], and it has been suggested as UK COVID-19 lockdown exit strategy [14]. There are also arguments that permanent lockdown can be theoretically replaced by mass testing supported by mobilizing a sufficient target population [15]. In addition, vaccination is considered the most likely strategy to exit the pandemic or transition to the endemic [16–19]. But the expectation of elimination of using vaccination has changed given the accumulating evidence of waning immunity, re-infection and new variant emergence [20]. The vaccines could achieve eliminating severe COVID-19, rather than eliminating SARS-CoV-2 infection [20]. Furthermore, there are arguments against Zero-COVID considering the inhumane health and social consequences for vulnerable groups [21]. Therefore, the policy choices may need to be assessed due to new circumstances and changing risk perceptions.

China has implemented the Zero-COVID policy from the first outbreak in January of 2020, and since then till early March, 2022, all of the local outbreaks caused by imported cases of wildtype have been controlled by this national zero-tolerance policy [22–25]. Once a positive case is confirmed, citywide multiple-rounds testing, tracing, and isolation (CTTI) are immediately implemented until no new infections are detected. The outbreak of Delta variant in Guangzhou [26] and the outbreak in Yangzhou [27] of China are another two successful examples of Zero-COVID policy. Nevertheless, given the population size and capacity of carrying out the CTTI, it is essential to consider many practical factors guaranteeing its success to reveal the dynamics of the CTTI strategy. It will not only allow to estimate the minimum rounds needed to eliminate the infections, but also contribute to the knowledge gap of allocation of insufficient public health resources when a CTTI is not possible. Although the zero-tolerance policy has been efficient in preventing new outbreaks, there are no available studies to quantify the mechanisms of the CTTI strategy and the associated health burden of containing the epidemic.

The first Omicron case was imported to the mainland of China on December 9, 2021, and due to its stronger transmissibility, the eradication of COVID-19 has become more difficult [28]. On March 1, 2022, Jilin Province announced its first confirmed case, a male who travelled back to Hunchun City. Subsequently, Jilin province experienced an explosive Omicron outbreak, with a total of 48,047 confirmed, and 16,519 of them are asymptomatic infections as of April 1, 2022 [29]. Besides the CTTI strategy, the tightened measures and the establishment of Fangcang shelter were carried out since Mid-March, 2022. Although aggressive CTTI strategy was implemented, there is debate whether COVID-19 eradication is possible to face the highly contagious Omicron variant [30].

We propose a Susceptible-Exposed-Asymptomatic-Infectious-Recovered (SEAIR) model that mimics the implementation of the CTTI Zero-COVID policy in curbing the local transmission in Fujian Province and Jilin Province, China, using the data of daily new confirmed cases, including symptomatic and asymptomatic infections (Fujian: September 10–October 9, 2021; Jilin: March 1–April 1, 2022) [29, 31]. We also include compartments to count for severe outcomes, such as death and hospitalizations. Our model assumes that all individuals in the susceptible, exposed, presymptomatic, and asymptomatic stages are traced and if in contact with infected individuals, held in isolation. The structure of our model allows us to identify not only the number of testing rounds needed to claim the elimination, but also the impact of early detection of asymptomatic and presymptomatic individuals. Our results confirm that citywide testing, contact tracing, isolation of infected individuals, most importantly tracing and isolations of first- and second-degree contacts of cases in time before further infections are induced, are crucial to ensure the success of the powerful approach, which will control not only the emerging outbreaks but also ongoing ones. We also explore the possibility of eradication with Omicron transmission using the CTTI strategy. We corroborate the importance of prompt enforcement of rigorous and continuous testing to ensure a rapid response to any possible new outbreak. Moreover, our findings suggest that the pandemic will last longer with possible recurrent outbreaks till a more effective vaccine and treatment medicine are available if the CTTI cannot be implemented.

## Methods

### Model overview

**Fig. 1:**
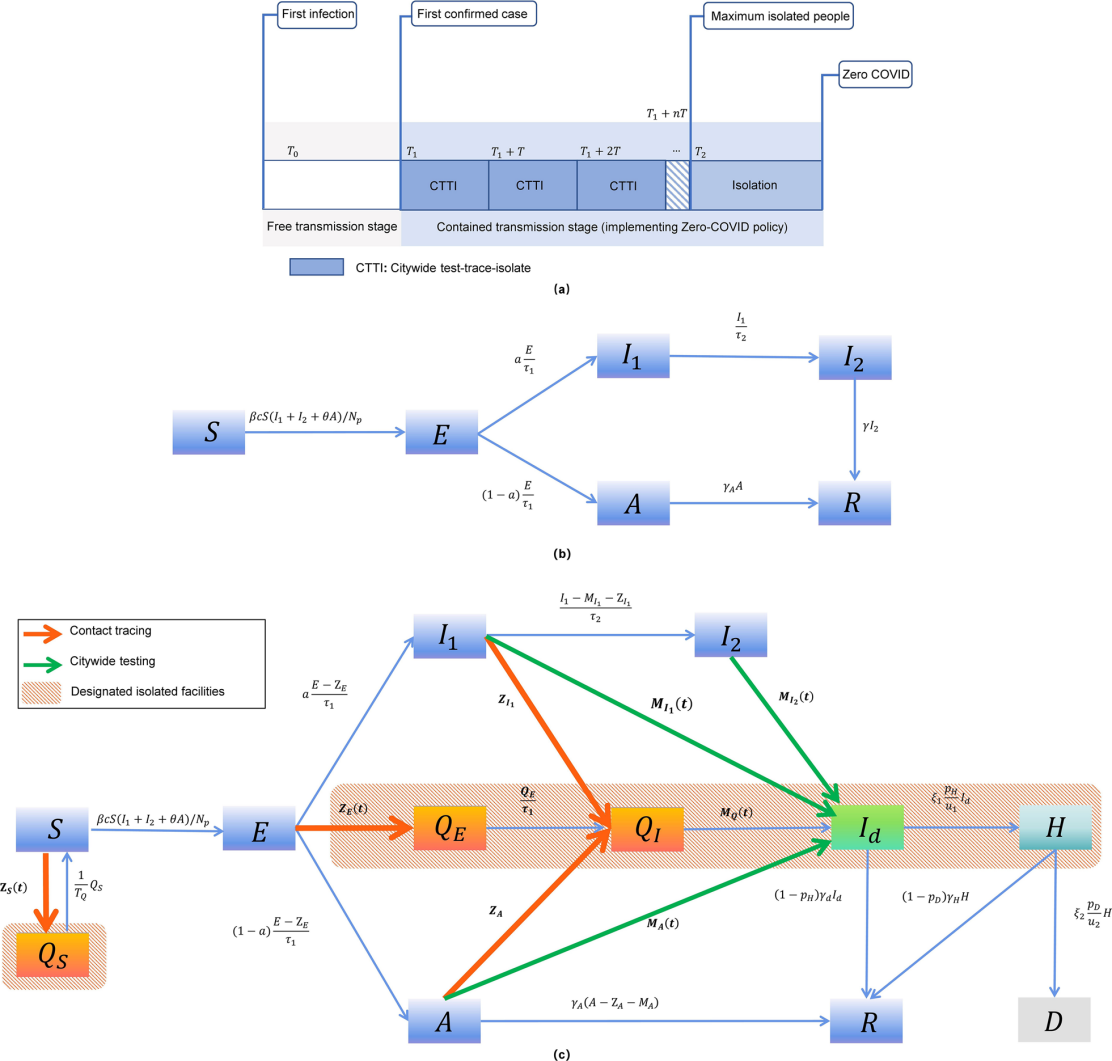
Illustration of the model process considering the implementation of CTTI in the Zero-COVID policy. **a** The schematic diagram of the model. The flow chart of the model in **b** free transmission stage **c** contained transmission stage. All individuals are in different states, Susceptible (*S*), Exposed (*E*), Infected (asymptomatic *A*, presymptomatic ${I}_{1}$ or with symptoms ${I}_{2}$), Recovered (*R*) or Deceased (*D*). If the confirmed cases were detected, we also include the diagnosed and isolated (${I}_{d}$) and the individuals admitted to ICU ($H$). The traced and isolated susceptible, exposed, asymptomatic and presymptomatic infected populations are described as ${Q}_{S}$, ${Q}_{E}$, ${Q}_{I}$, respectively. In the contained transmission stage, the arrow with orange color represents the process of contact tracing, while the green arrow shows the process of citywide testing and symptomatic testing. All the confirmed and traced individuals are isolated in the designated isolated facilities. The description of parameters shown in the flow diagram can be found in Table 1. ${T}_{0}$: initial time, ${T}_{1}:$ the date when the first infection is confirmed, $T$: the time needed to complete one round of citywide testing, ${T}_{2}$: the time when no new infections are detected. CTTI: citywide test-trace-isolate

We present our compartmental model incorporating the CTTI under the Zero-COVID policy as shown in Fig. 1. The spreading of the infection starts when a first individual is infected due to the case(s) imported into the community. Usually, it is not easy to identify the date when the first infection started. We denote such date as the initial time ${T}_{0}$, and denote the date when the first infection is confirmed as ${T}_{1}$, and refer this period as the “*Free transmission stage*”. Once the infection is confirmed, we consider the ideal scenario that the first round of CTTI is immediately conducted. From this point, we define the “*Contained transmission stage*” in which the Zero-COVID policy is implemented. We define $T$ as the time needed to complete one round of citywide testing. Multiple rounds of testing are conducted until the time ${T}_{2}$ when no new infections are detected. Within this stage, confirmed cases and their primary and secondary contacts are traced and isolated. In the process of implementing CTTI, it is unavoidable that susceptible individuals can be isolated too. It is important to observe that when no more individuals are isolated in 3–5 days, we claim that no infections are present in the community, which labels the elimination of the virus in the city.

We apply our model to estimate the number of testing rounds needed to achieve zero transmission for Fujian Province after calibrating the model using epidemiology data. We then estimate the rounds of citywide testing, cumulative cases, and in particular, the total number of isolated individuals under different free-transmission periods, testing capacities, and contact tracing efficiencies. In addition, we present the projections of the recent outbreak resulting from the Omicron transmission in Jilin province till April 30, 2022, under different scenarios. Furthermore, the uncertainty of parameters estimation is discussed by the sensitivity analysis.

### Study area and data sources

The first reported infection of the Delta variant of COVID-19 was detected in the routine testing of a primary school in Putian, a city in southeastern coastal of Fujian, on September 10, 2021, which was linked to the importation of cases from Singapore. It quickly ballooned into a province-wide outbreak [32], mostly in Putian, Xiamen, Zhangzhou, and Quanzhou (epidemic area, 22.2 million residents). The total number of reported infections reached over 100 in just 4 days [31]. The CTTI strategy was quickly put into action to halt the community spread. Hence, our deterministic transmission model was calibrated using publicly available data of the epidemic areas in Fujian Province (Xiamen, Putian, Quanzhou, and Zhangzhou), China, between September 10, 2021, and October 9, 2021, on daily confirmed cases by report date and the cumulative number of isolated people, collected from the website of Fujian Province Health Commission [31].

Here, we also apply our model to the recent Omicron outbreak that occurred in Jilin Province, China, using the data of daily new confirmed cases, including symptomatic and asymptomatic infections, from March 1, 2022, to April 1, 2022 [29].

### Contact tracing in China

The comprehensive and timely contact tracing serves as one of the crucial control measures in curbing the transmission of the COVID-19 epidemic. In February 2020, China rolled out its “health code” app nationwide (first in Zhejiang Province, then extend to national scale, see details in Additional file 1), which ensures the efficiency of the contact tracing as the status of the health QR code is updated with time according to the smartphone GPS data, including travel history, duration of time spent in the risky zone, and relationships to potential carriers [33, 34].

### Citywide testing in China

The pooled testing strategy is adopted to quickly find out infections and determine the risk level of the zone in the first round of testing. In the subsequent round of testing, the high-risk zones take the more precise 1:1 testing (place a single specimen in a test tube for testing), while the other zones implement 5:1 or 10:1 pooled testing [5]. During each round of testing, when a new case is detected, the person’s health QR code will immediately change to red. The contact tracing will be conducted based on the health QR code system, assigning red or yellow codes to the contacts. The risk level in the zones will be evaluated according to the number of newly detected populations after completing one round of testing.

The rolling out citywide testing (test everyone, symptom or not) as it battles the outbreak can be such a powerful tool for getting on top of the virus. In China, one round of citywide testing takes several days, depending on the population and testing capacity in the city, usually 2–3 days [35]. However, given the high transmissibility of Omicron variant, on March 22, 2022, *the third edition of Guidelines for Regional Nucleic Acid Testing* was issued with a specified requirement for completing citywide testing within 24 h [36].

### Model design

We investigate the effect of the Zero-COVID strategy using a deterministic compartmental Susceptible $S$—Exposed $E$—Asymptomatic $A$—Infectious ${I}_{1}$ (pre-symptomatic)—Infectious ${I}_{2}$ (symptomatic)—Recovered *R*—Deceased *D* model framework. When the cases are detected, we also include the diagnosed and isolated ${I}_{d}$ and the individuals admitted into ICU $H$. The contact tracing process is also modeled by defining ${Q}_{S}$, ${Q}_{E}$, ${Q}_{I}$ as the number of traced and isolated susceptible, exposed, and asymptomatic or presymptomatic infected populations, respectively. Here, asymptomatic infections refer to infected individuals who have no symptoms during the whole infection period. The presymptomatic is the prodromal phase of the symptomatic infections, and it has no symptoms but will develop symptoms in the following days. Note that we omit demographic components, such as immigration, birth, and natural death. And we consider the Delta variant in the Fujian case and the Omicron variant in the Jilin case.

We model the transmission process starting with local people infected by the imported virus and ending with zero COVID cases. Two stages before and after the detection of the first confirmed cases at the time ${T}_{1}$, defined as free transmission stage and contained transmission stage, are considered (Fig. 1). In the period of free transmission stage $[{T}_{0},{T}_{1}]$, there is no confirmed case. The local transmission occurs without any intervention measures. Then, the first round of testing should be performed immediately at ${T}_{1}$. For $t\in [{T}_{1},{T}_{1}+nT]$, where $n$ is the number of rounds of citywide testing needed to eliminate the cases. The number of daily new diagnosed cases at time $t$ is denoted as ${M}_{{I}_{d}}(t)$, which is the sum of daily new diagnosed asymptomatic ${M}_{A}\left(t\right)$ and presymptomatic ${M}_{{I}_{1}}\left(t\right)$ infections from citywide testing, as well as symptomatic infections ${M}_{{I}_{2}}\left(t\right)$ from symptomatic testing. Also, at the time ${T}_{1}$, the primary contacts of confirmed cases are immediately traced and isolated, but we ignore the tracing of secondary contacts of the confirmed cases at the time ${T}_{1}$ due to the effort needed for the initial contact tracing. After that time, all the primary and secondary contacts are efficiently traced and isolated. The contact tracing process for ${M}_{{I}_{d}}(t)$ results in the move-out of traced susceptible (${\rm Z}_{S}\left(t\right)$), exposed (${\rm Z}_{E}\left(t\right)$), presymptomatic infectious (${\rm Z}_{{I}_{1}}\left(t\right)$), and asymptomatic infectious (${\rm Z}_{A}\left(t\right)$) individuals and their sum is the total traced population of confirmed cases from citywide testing and symptomatic testing at time $t$, i.e. ${\rm Z}_{CT}\left(t\right)$.

We further denote $Q\left(t\right)$ as the total number of isolated populations until day $t$ due to testing and contact tracing, which includes all the traced and confirmed people at that time, i.e., $Q\left(t\right)={\int }_{{T}_{1}}^{t}{M}_{{I}_{d}}\left(t\right)+{\rm Z}_{CT}\left(t\right) dt$. Here we emphasize $Q\left(t\right)$ is an effective measure of Zero-COVID policy. When $Q\left(t\right)$ does not increase and reach its maximum, i.e., $Q{\left(t\right)}^{^{\prime}}=0$, it means we successfully find out all the infected population and cut off the transmission route by putting them into designated isolation facilities. Also, it is equivalent to the fact that there is no new confirmed case from citywide testing. Hence, after $n$ rounds of citywide testing, when there is no new confirmed case from citywide testing or $Q{\left(t\right)}^{^{\prime}}=0$, the zero-covid policy succeeds. Otherwise, we conduct the next round of citywide testing.

There are three separative testing in the contained transmission stage: symptom-driven testing, citywide testing, and the testing for primary and secondary contacts at the designated isolation facilities. And $\eta$ is the testing efficiency towards asymptomatic infections $A$ and presymptomatic infections ${I}_{1}$. The adherence rate of citywide testing and symptom-driven testing is assumed to be ${\alpha }_{1}$, ${\alpha }_{2}$, respectively. Also, we introduce ${M}_{Q}\left(t\right)$ as the number of daily new confirmed individuals at designated isolation facilities. Testing for the traced population is implemented frequently, and all the tested people with positive results (${M}_{Q}\left(t\right)$) will transfer to the confirmed class ${I}_{d}$.

#### Contact tracing for confirmed cases

We define ${c}_{d}$ as the average number of primary contacts per confirmed case within 2 days before the onset of symptoms or collecting samples. We consider it is proportional to the number of contacts per individual per day $c$ so that it can be expressed as

$${c}_{d}={p}_{c}\left(2c-{n}_{H}\right),$$

where ${0\le p}_{c}\le 1$ is the efficiency of contact tracing and ${n}_{H}$ is the average number of household sizes. We also introduce ${c}_{S}$ as the average number of secondary contacts of confirmed cases within 2 days before the onset of symptoms or collecting samples. We assume that there is no overlapping of primary contacts and secondary contacts. Then we have

$${c}_{s}={c}_{d}({c}_{d}-1).$$

Therefore, the number of primary contacts and secondary contacts of newly diagnosed infections from citywide testing at time $t$ is

$${\rm Z}_{CT}\left(t\right)=\left({c}_{d}+{c}_{s}\right){M}_{{I}_{d}}\left(t\right).$$

We assume that the symptomatic infections are already detected by symptom-driven testing. Hence, the traced population is composed of susceptible, exposed, asymptomatic, and presymptomatic individuals. The number of the traced individuals that are exposed is denoted as $\left({\rm Z}_{E}\left(t\right)\right)$ depends on: (a) the transmission probability in each contact $(\beta )$, (b) the number of primary and secondary contacts per diagnosed case (${c}_{d}$ and ${c}_{s}$), (c) the probability of being exposed individuals $\left(\frac{{\tau }_{1}}{{\tau }_{1}+{\tau }_{2}}\right)$, and (d) the number of diagnosed cases from citywide testing, which is

$${\rm Z}_{E}\left(t\right)={M}_{{I}_{d}}\left(t\right)\left({c}_{d}\beta +{c}_{s}{\beta }^{2}\right)\frac{{\tau }_{1}}{{\tau }_{1}+{\tau }_{2}}.$$

Similarly, we can obtain the number of traced presymptomatic infections, which is

$${\rm Z}_{{I}_{1}}\left(t\right)={M}_{{I}_{d}}\left(t\right)\left({c}_{d}\beta +{c}_{s}{\beta }^{2}\right)\frac{{\tau }_{2}a}{{\tau }_{1}+{\tau }_{2}}.$$

The number of traced asymptomatic infections is

$${\rm Z}_{A}\left(t\right)={M}_{{I}_{d}}\left(t\right)\left({c}_{d}\beta +{c}_{s}{\beta }^{2}\right)\frac{{\tau }_{2}\left(1-a\right)}{{\tau }_{1}+{\tau }_{2}}.$$

The number of traced susceptible individuals is

$${\rm Z}_{s}\left(t\right)={M}_{{I}_{d}}\left(t\right)\left[{c}_{d}\left(1-\beta \right)+{c}_{s}\left(1-{\beta }^{2}\right)\right].$$

Therefore, the traced population of confirmed cases from citywide testing are the sum of the above, i.e.,

$${\rm Z}_{CT}\left(t\right)={\rm Z}_{s}\left(t\right)+{\rm Z}_{{I}_{1}}\left(t\right)+{\rm Z}_{E}\left(t\right)+{\rm Z}_{A}\left(t\right).$$

#### Testing for traced population

Testing for the traced population is conducted separately and has a higher frequency compared with citywide testing. The number of detected people from testing for those traced population at time $t$ ${M}_{Q}(t)$ only depends on the testing accuracy $\eta$, which yields

$${M}_{Q}\left(t\right)=\eta {Q}_{I}\left(t\right).$$

We do not consider the contact tracing for ${M}_{Q}$, because (1) the primary and secondary contacts of ${M}_{Q}$ are third or fourth contacts of the original confirmed case and have less probability of being infected; (2) ${M}_{Q}$ may be exposed infections when they are traced and unable to transmit the virus before being traced; (3) even if ${M}_{Q}$ is asymptomatic or presymptomatic infections when they are traced, their infection can be detected from subsequent testing; (4) when ${M}_{Q}$ are traced, they are isolated and do not contribute to further transmission.

#### The symptomatic testing strategy

The symptomatic infectious individuals ${I}_{2}$ get tested at time $t$ are

$${M}_{{I}_{2}}\left(t\right)=\frac{{\alpha }_{2}}{{u}_{{I}_{2}}}{I}_{2}\left(t\right),$$

where ${\alpha }_{2}$ is the adherence rate to the symptomatic testing and ${u}_{{I}_{2}}$ is the average day from symptom onset to confirmation.

#### The citywide testing strategy

The number of individuals who will get tested in this current round of citywide testing at time $t$ can be defined as ${\rm Z}_{PCR}(t)$, which is

$${\rm Z}_{PCR}\left(t\right)=\frac{1}{T}\left(N-{\int }_{{T}_{1}}^{t}{\rm Z}_{CT}\left(t\right)dt-{\int }_{{T}_{1}}^{t}{M}_{{I}_{d}}\left(t\right)dt\right),$$

where $N$ is the total population in the city. From the citywide testing, the asymptomatic, presymptomatic can be detected. Also, the number of asymptomatic infectious individuals $A$ detected in this round of citywide testing at time $t$ (${M}_{A}\left(t\right)$) depends on the following factors, the proportion of asymptomatic infectious individuals in the tested population, the number of individuals who will get tested in this round of testing at time $t$, the testing efficiency for the asymptomatic infectious population, and the adherence rate of the population participating citywide testing.

$${M}_{A}\left(t\right)=\frac{A\left(t\right)}{{N}_{T}\left(t\right)}{\rm Z}_{PCR}\left(t\right)\eta {\alpha }_{1},$$

where $${N}_{T}\left(t\right)=S\left(t\right)+E\left(t\right)+A\left(t\right)+{I}_{1}\left(t\right).$$

Similarly, we can obtain that the presymptomatic infectious individuals ${I}_{1}$ removed from citywide testing at time $t$, which are

$${M}_{{I}_{1}}\left(t\right)=\frac{{I}_{1}\left(t\right)}{{N}_{T}\left(t\right)}{\rm Z}_{PCR}\left(t\right)\eta {\alpha }_{1}.$$

Therefore, the number of daily new diagnosed cases at time $t$ is the sum of tested people from citywide testing and symptomatic testing, which yields

$${M}_{{I}_{d}}={M}_{A}\left(t\right)+{M}_{{I}_{1}}\left(t\right)+{M}_{{I}_{2}}\left(t\right).$$

#### The free transmission stage model

The flow chart of the model in the free transmission stage is presented in Fig. 1b. And we have the following model

$$\left\{\begin{array}{l}{S}^{^{\prime}}=-\beta cS\left({I}_{1}+\theta A+{I}_{2}\right)/{N}_{p} ,\\ {E}^{^{\prime}}=\beta cS\left({I}_{1}+\theta A+{I}_{2}\right)/{N}_{p}-\frac{1}{ {\tau }_{1}}E,\\ {I}_{1}^{^{\prime}}=\frac{a}{ {\tau }_{1}}E-{\frac{1}{{\tau }_{2}}I}_{1},\\ {A}^{^{\prime}}=\frac{1-a}{ {\tau }_{1}}E-{\gamma }_{A}A,\\ {I}_{2}^{^{\prime}}=\frac{1}{{\tau }_{2}}{I}_{1}-\gamma {I}_{2},\\ {R}^{^{\prime}}={\gamma }_{A}A+\gamma {I}_{2}.\end{array}\right.$$

where ${N}_{P}\left(t\right)=S\left(t\right)+E\left(t\right)+A\left(t\right)+{I}_{1}\left(t\right)+{I}_{2}\left(t\right)$, and with the initial state

$$S\left({T}_{0}\right)={S}_{0},E\left({T}_{0}\right)={E}_{0},{I}_{1}\left({T}_{0}\right)=0,A\left({T}_{0}\right)=0,{I}_{2}\left(0\right)=0,R=0$$

where $${E}_{0}\ne 0$$, and $${S}_{0}=N-{E}_{0}$$.

At time ${T}_{1}$, we have state values $\left\{S\left({T}_{1}\right),E\left({T}_{1}\right),{I}_{1}\left({T}_{1}\right),A\left({T}_{1}\right),{I}_{2}\left({T}_{1}\right),R\left({T}_{1}\right)\right\}.$ If the free transmission stage is quite short, there are no recovered individuals during this stage, i.e., $R\left(t\right)=0$ for $t\in [{T}_{0},{T}_{1}].$

#### The contained transmission stage model with the CTTI strategy

Based on our previous analysis, we have the model in the contained transmission stage where the CTTI strategy is conducted, and its flow chart is shown in Fig. 1c. The initial values at ${T}_{1}$ should be recalculated as there are detected cases ${M}_{{I}_{d}}({T}_{1})$, who may have developed symptoms or not. Hence, the initial conditions at ${T}_{1}$ are the results after moving out the traced susceptible population ${\rm Z}_{s}\left({T}_{1}\right)$, exposed population ${\rm Z}_{E}\left({T}_{1}\right)$, presymptomatic population ${\rm Z}_{{I}_{1}}\left({T}_{1}\right)$, asymptomatic population ${\rm Z}_{A}\left({T}_{1}\right)$, and detected cases ${M}_{{I}_{d}}\left({T}_{1}\right)+{M}_{Q}\left({T}_{1}\right)$, where

$${\rm Z}_{A}\left({T}_{1}\right)={M}_{{I}_{d}}\left({T}_{1}\right){c}_{d}\beta \frac{{\tau }_{2}(1-a)}{{\tau }_{1}+{\tau }_{2}},$$

$${\rm Z}_{{I}_{1}}\left({T}_{1}\right)={M}_{{I}_{d}}\left({T}_{1}\right){c}_{d}\beta \frac{{\tau }_{2}a}{{\tau }_{1}+{\tau }_{2}},$$

$${\rm Z}_{E}\left({T}_{1}\right)={M}_{{I}_{d}}\left({T}_{1}\right){c}_{d}\beta \frac{{\tau }_{1}}{{\tau }_{1}+{\tau }_{2}},$$

$${\rm Z}_{S}\left({T}_{1}\right)={M}_{{I}_{d}}\left({T}_{1}\right){c}_{d}\left(1-\beta \right).$$

Then the initial state at ${T}_{1}$ is

$$\left\{{S}_{{T}_{1}},{E}_{{T}_{1}},{{I}_{1}}_{{T}_{1}},{A}_{{T}_{1}},{I}_{{{2}_{T}}_{1}},{Q}_{{s}_{{T}_{1}}},{Q}_{{E}_{{T}_{1}}},{Q}_{{I}_{{T}_{1}}},{I}_{{d}_{{T}_{1}}},{H}_{{T}_{1}},{R}_{{T}_{1}},{D}_{{T}_{1}},{Q}_{{T}_{1}}\right\},$$

where $${S}_{{T}_{1}}=S\left({T}_{1}\right)-{\rm Z}_{s}\left({T}_{1}\right),$$ $${E}_{{T}_{1}}=E\left({T}_{1}\right)-{\rm Z}_{E}\left({T}_{1}\right),$$ $${{I}_{1}}_{{T}_{1}}={I}_{1}\left({T}_{1}\right)-{M}_{{I}_{1}}\left({T}_{1}\right)-{\rm Z}_{{I}_{1}}\left({T}_{1}\right),$$

$${A}_{{T}_{1}}=A\left({T}_{1}\right)-{M}_{A}\left({T}_{1}\right)-{\rm Z}_{A}\left({T}_{1}\right),{{I}_{{{2}_{T}}_{1}}=I}_{2}\left({T}_{1}\right)-{M}_{{I}_{2}}\left({T}_{1}\right),{Q}_{{s}_{{T}_{1}}}={\rm Z}_{S}\left({T}_{1}\right),{Q}_{{E}_{{T}_{1}}}={\rm Z}_{E}\left({T}_{1}\right),$$

$${Q}_{{I}_{{T}_{1}}}={\rm Z}_{A}\left({T}_{1}\right)+{\rm Z}_{{I}_{1}}\left({T}_{1}\right),{I}_{{d}_{{T}_{1}}}={M}_{{I}_{1}}\left({T}_{1}\right)+{M}_{A}\left({T}_{1}\right)+{M}_{{I}_{2}}\left({T}_{1}\right)+{M}_{Q}\left({T}_{1}\right),{H}_{{T}_{1}}=0,{R}_{{T}_{1}}=R\left({T}_{1}\right),$$

$${D}_{{T}_{1}}=0,{Q}_{{T}_{1}}={\rm Z}_{S}\left({T}_{1}\right)+{\rm Z}_{E}\left({T}_{1}\right)+{\rm Z}_{{I}_{1}}\left({T}_{1}\right)+{\rm Z}_{A}\left({T}_{1}\right)+{M}_{{I}_{1}}\left({T}_{1}\right)+{M}_{A}\left({T}_{1}\right)+{M}_{{I}_{2}}\left({T}_{1}\right).$$

Starting from ${T}_{1}$, multi citywide testing is conducted. At the end of $n$th ($n\ge 1$) round of testing, we have the value of each state, i.e., $\left\{S\left({T}_{1}+nT\right),E\left({T}_{1}+nT\right),A\left({T}_{1}+nT\right),{I}_{1}\left({T}_{1}+nT\right),{I}_{2}\left({T}_{1}+nT\right),{I}_{d}\left({T}_{1}+nT\right),{Q}_{S}\left({T}_{1}+nT\right),{Q}_{E}\left({T}_{1}+nT\right),{Q}_{I}\left({T}_{1}+nT\right),H\left({T}_{1}+nT\right),R\left({T}_{1}+nT\right),D\left({T}_{1}+nT\right),Q\left({T}_{1}+nT\right)\right\}.$ From our analysis, if the total number of isolated people reaches the maximum, i.e.,

$$Q{\left({T}_{1}+nT\right)}^{^{\prime}}=0$$

which is equivalent to ${M}_{{I}_{d}}\left(t\right)=0$, we achieve the goal of zero covid cases. And if it is not zero, we move on to the next round of testing.

So, the model on the $t\in \left[{T}_{1}+\left(n-1\right)T,{T}_{1}+nT\right]$ ($n\ge 1$) is

$$\left\{ \begin{gathered} S^\prime = - \beta cS\left( {{I_1} + \theta A + {I_2}} \right){N_p} - {{\text{Z}}_S}\left( t \right) + \frac{1}{{{T_Q}}}{Q_S}, \hfill \\ E^\prime = \beta cS\left( {{I_1} + \theta A + {I_2}} \right){N_p} - \frac{1}{{{\tau _1}}}(E - {{\text{Z}}_E}) - {{\text{Z}}_E}\left( t \right), \hfill \\ I_1^\prime = \frac{a}{{{\tau _1}}}(E - {Z_E}) - \frac{1}{{{\tau _2}}}\left( {{I_1} - {{\text{Z}}_{{I_1}}}\left( t \right) - {M_{{I_1}}}\left( t \right)} \right) - {{\text{Z}}_{{I_1}}}\left( t \right) - {M_{{I_1}}}\left( t \right), \hfill \\ A^\prime = \frac{{1 - a}}{{{\tau _1}}}(E - {Z_E}) - {{\text{Z}}_A}\left( t \right) - {M_A}\left( t \right) - {\gamma _A}\left( {A - {{\text{Z}}_A}\left( t \right) - {M_A}\left( t \right)} \right), \hfill \\ I_2^\prime = \frac{1}{{{\tau _2}}}\left( {{I_1} - {{\text{Z}}_{{I_1}}}\left( t \right) - {M_{{I_1}}}\left( t \right)} \right) - {M_{{I_2}}}\left( t \right), \hfill \\ Q_S^\prime = {{\text{Z}}_S}\left( t \right) - \frac{1}{{{T_Q}}}{Q_S}, \hfill \\ Q_E^\prime {\text{ = }}{{\text{Z}}_{\text{E}}}\left( {\text{t}} \right) - \frac{{\text{1}}}{{{\tau _{\text{1}}}}}{{\text{Q}}_{\text{E}}}, \hfill \\ {\text{Q}}_I^\prime {\text{ = }}{{\text{Z}}_{{{\text{I}}_{\text{1}}}}}\left( {\text{t}} \right){\text{ + }}{{\text{Z}}_{\text{A}}}\left( {\text{t}} \right){\text{ + }}\frac{{\text{1}}}{{{\tau _{\text{1}}}}}{{\text{Q}}_{\text{E}}} - {{\text{M}}_{\text{Q}}}\left( {\text{t}} \right), \hfill \\ {\text{I}}_d^\prime {\text{ = }}{{\text{M}}_{{{\text{I}}_{\text{1}}}}}\left( {\text{t}} \right){\text{ + }}{{\text{M}}_{\text{A}}}\left( {\text{t}} \right){\text{ + }}{{\text{M}}_{{{\text{I}}_{\text{2}}}}}\left( {\text{t}} \right){\text{ + }}{{\text{M}}_{\text{Q}}}\left( {\text{t}} \right) - {\gamma _{\text{d}}}\left( {{\text{1}} - {{\text{p}}_{\text{H}}}} \right){{\text{I}}_{\text{d}}} - {\xi _{\text{1}}}\frac{{{{\text{p}}_{\text{H}}}}}{{{{\text{u}}_{\text{1}}}}}{{\text{I}}_{\text{d}}}, \hfill \\ {\text{H}}^\prime {\text{ = }}{\xi _{\text{1}}}\frac{{{{\text{p}}_{\text{H}}}}}{{{{\text{u}}_{\text{1}}}}}{{\text{I}}_{\text{d}}} - {\xi _{\text{2}}}\frac{{{{\text{p}}_{\text{D}}}}}{{{{\text{u}}_{\text{2}}}}}{\text{H}} - {\gamma _{\text{H}}}({\text{1}} - {{\text{p}}_{\text{D}}}){\text{H}}, \hfill \\ {\text{R}}^\prime {\text{ = }}{\gamma _{\text{A}}}\left( {{\text{A}} - {{\text{Z}}_{\text{A}}}\left( {\text{t}} \right) - {{\text{M}}_{\text{A}}}\left( {\text{t}} \right)} \right){\text{ + }}{\gamma _{\text{d}}}\left( {{\text{1}} - {{\text{p}}_{\text{H}}}} \right){{\text{I}}_{\text{d}}}{\text{ + }}{\gamma _{\text{H}}}({\text{1}} - {{\text{p}}_{\text{D}}}){\text{H}}, \hfill \\ {\text{D}}^\prime {\text{ = }}{\xi _{\text{2}}}\frac{{{{\text{p}}_{\text{D}}}}}{{{{\text{u}}_{\text{2}}}}}{\text{H}}, \hfill \\ {\text{Q}}^\prime {\text{ = }}{{\text{Z}}_{\text{S}}}\left( {\text{t}} \right){\text{ + }}{{\text{Z}}_{\text{E}}}\left( {\text{t}} \right){\text{ + }}{{\text{Z}}_{{{\text{I}}_{\text{1}}}}}\left( {\text{t}} \right){\text{ + }}{{\text{Z}}_{\text{A}}}\left( {\text{t}} \right){\text{ + }}{{\text{M}}_{{{\text{I}}_{\text{1}}}}}\left( {\text{t}} \right){\text{ + }}{{\text{M}}_{\text{A}}}\left( {\text{t}} \right){\text{ + }}{{\text{M}}_{{{\text{I}}_{\text{2}}}}}\left( {\text{t}} \right). \hfill \\ \end{gathered} \right.$$

### Parameters estimation

We estimate the following parameters, including the probability of transmission per contact and the contact rate in each stage, by using a Monte-Carlo-based Bayesian modeling framework [37, 38]. We sample 100,000 parameter vectors from the uniform prior distribution (Additional file 1: Table S1), and we choose the 1000 best-fitting parameter vectors based on the modified normalized mean square error (NMSE) indicator by comparing data and model predictions of cumulative confirmed cases and cumulative isolated individuals. The median and 95% confidence interval (*CI*) of the estimated parameters are obtained through 1000 best-fitting parameters vectors (Additional file 1: Table S1). The estimated parameter values and other parameters obtained from the literature for the case of Fujian are presented in Table 1. The given initial conditions and parameters used in the Jilin case can be found in the Additional file 1: Table S2 and S3. In the scenarios analysis, we show the simulation results using the parameter vectors with the minimum NMSE indicators.

**Table 1:** Model variables and parameters

Notation	Description	Value at ${t}_{0}$	Sources
*Variables and their initial values*			
$S(t)$	The number of susceptible individuals at day $t$	22,211,296	Assumed
$E(t)$	The number of exposed individuals at day $t$	1	Assumed
$A(t)$	The number of asymptomatic infectious at day $t$	0	Assumed
${I}_{1}(t)$	The number of presymptomatic infectious individuals at day $t$	0	Assumed
${I}_{2}(t)$	The number of symptomatic infectious individuals at day $t$	0	Assumed
${I}_{d}(t)$	The number of confirmed and isolated individuals at day t	0	Assumed
$R(t)$	The number of recovered individuals at day t	0	Assumed
${Q}_{S}(t)$	The number of traced and quarantined susceptible individuals at day $t$	–	
${Q}_{E}(t)$	The number of traced and quarantined exposed individuals at day $t$	–	
${Q}_{I}(t)$	The number of traced and quarantined presymptomatic and asymptomatic infectious individuals at day $t$	–	
$H(t)$	The number of severe patients that need intensive health care (ICU)	–	
$D\left(t\right)$	The number of deceased individuals at day t	–	
${I}_{d}({T}_{1})$	The number of confirmed and isolated individuals at day ${T}_{1}$	5	Data [31]
*Parameters for COVID-19 in Fujian*			
${\tau }_{1}$	Average time spent in the exposed state, $E$, days, for Delta variant	2	Ref [49]
${\tau }_{2}$	Average time spent in the presymptomatic infected state ${I}_{1}$, days, for Delta variant	2	Ref [50]
$a$	Proportion of infected people who will develop symptoms	0.8	Ref [51]
$b$	Infectiousness of asymptomatic individuals compared to symptomatic infections	0.75	Ref [12]
$\eta$	The testing efficiency towards asymptomatic infections $A$ and presymptomatic infections ${I}_{1}$	0.67	Ref [52]
${\xi }_{1}$	The risk increment of ICU requirement for Delta variant compared to the wildtype	2.34	Ref [53]
${\xi }_{2}$	The risk increment of death for Delta variant compared to the wildtype	1.32	Ref [53]
${p}_{D}$	Proportion of severe patients in ICU died	18%	Ref [54]
${u}_{1}$	Days for developing severe symptoms and need ICU care after diagnosed, days	7–12	Ref [55]
${u}_{2}$	Length of stay for severe patients in ICU before died, days	15	Ref [56]
${u}_{{I}_{2}}$	The average days from symptom onset to diagnosis in Fujian, days	2	Ref [57]
${\gamma }_{A}$	Recovery rate of asymptomatic infectious individuals, days^−1^	1/6	Ref [58]
$\gamma$	Recovery rate of symptomatic infectious individuals, days^−1^	Stage 1: 0	Assumed
${\gamma }_{d}$	Recovery rate of non-ICU patients, days^−1^	1/7	Ref [58]
${\gamma }_{H}$	Recovery rate of ICU patients, days^−1^	1/11	Ref [59]
${\alpha }_{1}$	The adherence rate of citywide testing in Fujian	1	Ref [35]
${\alpha }_{2}$	The adherence rate of symptom-driven testing in Fujian	1	Ref [5]
${p}_{c}$	The percentage of contact tracing in Fujian	1	Ref [5]
$P$	Total number of populations in epidemic areas (Xiamen, Putian, Quanzhou, and Zhangzhou) in Fujian Province	22,211,297	Data [60]
${T}_{0}$	Time when the virus imported into epidemic areas in Fujian Province	September 2	Assumed
${T}_{1}$	Time when the first confirmed cases detected	September 10	Data [31]
${T}_{Q}$	The period of isolation for those traced individuals, days	28	Ref [5]
$T$	The time needed to complete one round of citywide test, days	2–3	Ref [35]
${Z}_{CT}(t)$	The number of people traced who has primary and secondary contacts of suspected or confirmed cases of COVID-19 at time t	–	
${Z}_{S}(t)$	Daily new isolated susceptible individuals due to contact tracing	–	
${Z}_{E}(t)$	Daily new isolated exposed individuals due to contact tracing	–	
${Z}_{A}(t)$	Daily new isolated asymptomatic individuals due to contact tracing	–	
${Z}_{{I}_{1}}\left(t\right)$	Daily new isolated presymptomatic infected individuals due to contact tracing	–	
${N}_{PCR}(t)$	The number of individuals conducting citywide testing at time t	–	
${M}_{A}\left(t\right)$	Daily new diagnosed asymptomatic infectious individuals from citywide testing	–	
${M}_{{I}_{1}}\left(t\right)$	Daily new diagnosed presymptomatic infectious individuals from citywide testing	–	
${M}_{{I}_{2}}\left(t\right)$	Daily new diagnosed symptomatic infectious individuals from symptom-driven testing	–	
${M}_{{I}_{d}}\left(t\right)$	Daily new diagnosed cases from citywide testing and symptom-driven testing	–	
*Estimated parameters*			
$\beta$	Probability of transmission per contact	0.0611 (95% *CI*: 0.0603–0.0804)	
$c$	The number of contacts per individual per day in each stage	Stage 1: 15.0220 (95% *CI*: 11.6387–15.4681) Stage 2: 8.7054 (95% *CI*: 7.7021–11.4798)	
${T}_{2}$	Time when the cumulative isolated population reached the maximum in Fujian Province	October 9	

### Scenarios and sensitivity analysis

We investigate the effect of different degrees of the CTTI policy, comparing processes such as contact tracing and testing resources. Also, the impacts of early detection, test capacity, and contact tracing efficiency on the transmission are examined. The minimum rounds of citywide testing to eliminate the COVID cases in different scenarios are compared, as well as the number of daily new confirmed cases and the total number of isolated individuals. We also explore the possibility of the CTTI strategy to control the ongoing outbreak driven by the new contagious Omicron variant. We present the projections of the Jilin outbreak till April 30, 2022, under the scenarios with the estimated parameter from data fitting and the escalated CTTI strategy. We conduct analyses using MATLAB (R2020a) [39]. Data and code are available from https://github.com/YTan94/ZeroCOVID_.

To study the sensitivity of the CTTI policy-related parameters on the model outputs, such as cumulative cases and isolated people, we use the Latin Hypercube Sampling/Partial Rank Coefficient (LHS/PRCC) method [40], generating 1000 samples for each parameter. The ranges used in the LHS are reported in Table 2. The parameters with a PRCC of magnitude greater than or equal to 0.5 were considered significant in the cumulative cases and isolated people [41].

**Table 2:** Parameters range for the Latin Hypercube Sampling (LHS) in the sensitivity analysis

Parameters	Distribution	Range
Free transmission period (${T}_{1}-{T}_{0}$)	Uniform	$\left[5, 12\right]$
Tracing percentage (${p}_{c}$)	Uniform	$\left[0.4, 1\right]$
Days per round of testing ($T$)	Uniform	$\left[1, 7\right]$
Citywide testing adherence rate (${\alpha }_{1}$)	Uniform	$\left[0.4, 1\right]$
Days from symptom onset to diagnosis (${U}_{{I}_{2}}$)	Uniform	$\left[1, 5\right]$
Testing efficiency ($\eta$)	Uniform	$\left[0.1, 1\right]$

## Results

### CTTI efficiently eliminates the recent COVID outbreak in Fujian, China

**Fig. 2:**
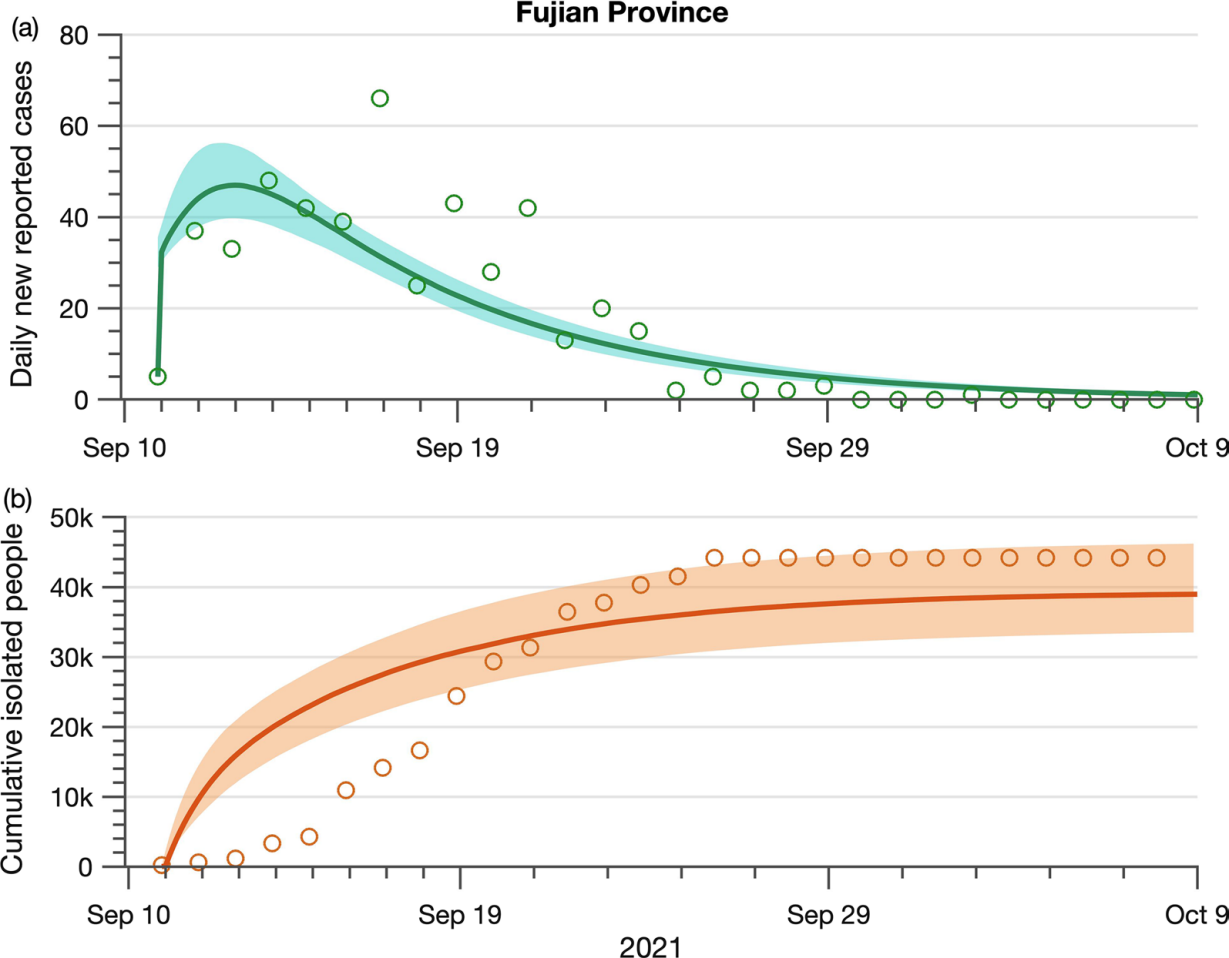
The COVID-19 daily incidence (**a**) and cumulative isolated people over time (**b**). Model validation and simulations of COVID-19 daily new cases (green color) and cumulative isolated individuals (orange color) in the epidemic area of Fujian, China, from September 10 to October 9, 2021. The circles represent observed data. The solid lines and shaded areas represent the median and 95% confidence interval of model simulations

Here, we investigate how the CTTI strategy Correlation works in controlling the local outbreak using the outbreak in September 2021 in Fujian as a case study. The model calibration results using the data from the Fujian outbreak are shown in Fig. 2. We predict that the provincial outbreak will end on October 9, 2021, with a total of 491 (95% *CI*: 430–567) infections after 10 (95% *CI*: 9–11) rounds of citywide testing.

We also observe that the epidemic progression is confined, along with the increased total number of isolated people. When the cumulative isolated people reach a plateau, all the possible infectious people are isolated, and local cases are eliminated. The number of cumulative isolated people can serve as a novel yet effective indicator to measure the effort of the public health sector to eradicate the local outbreak, achieving zero community transmission. With the aggressive Zero-COVID policy, the containment of the Delta virus in Fujian will only take 30 days. This result signals the efficiency of the CTTI policy resulting in a quick curbing of the local transmission.

### The application of CTTI if early detection is delayed

**Fig. 3:**
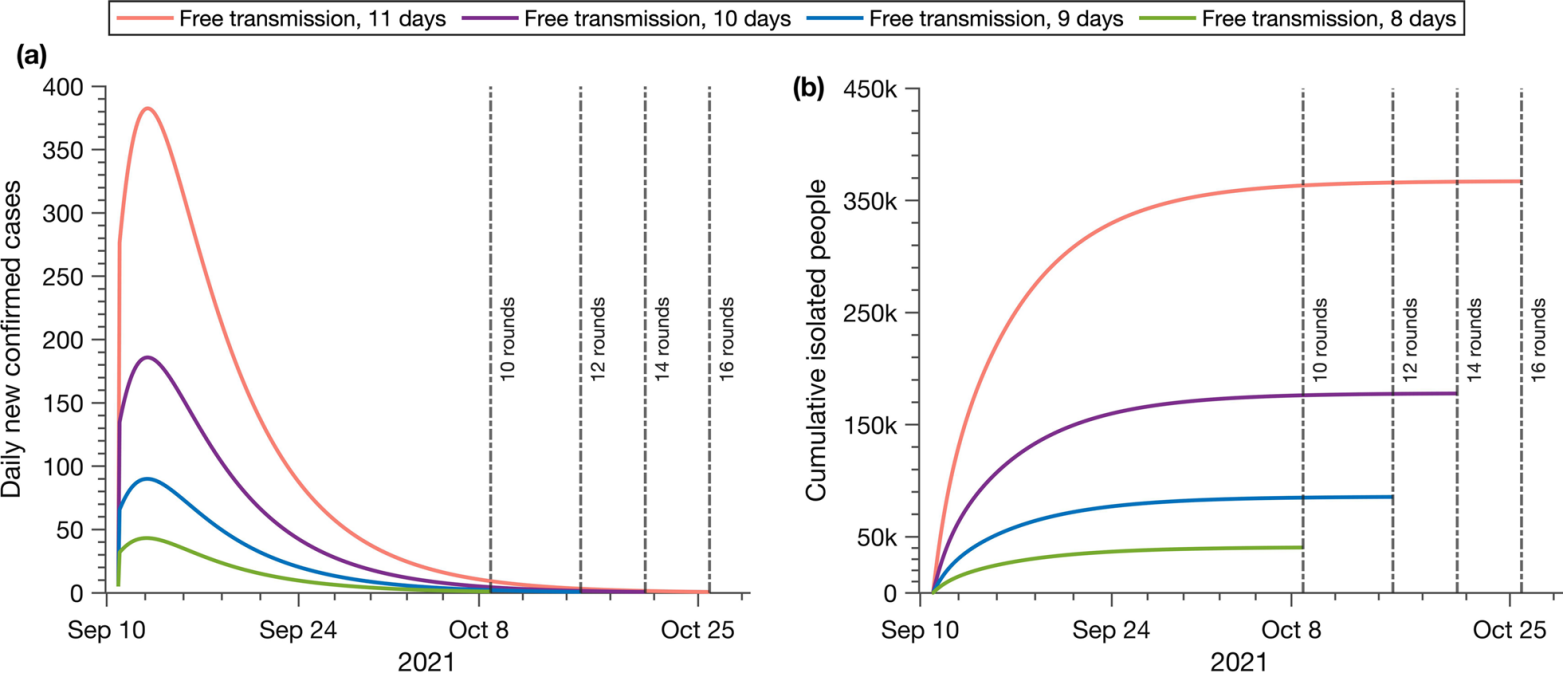
The effect of early detection on the containment of the COVID-19 epidemic. The daily new confirmed cases and the rounds of citywide testing (**a**) and the cumulative isolated individuals (**b**) are reported under different lengths of free transmission stage, 8 days (green color), 9 days (blue color), 10 days (purple color), and 11 days (light red color)

We investigate the impact of the CTTI strategy if early detection to confirm the first case is missed or postponed (Fig. 3). We assume the time of one round of citywide testing to be 3 days with an average of 7.4 million tests per day, and 100% of contacts are traced, referred to as the perfect CTTI. We observe that the early detection to identify the first local case is crucial for the elimination of the transmission. The later the first case is detected, the more resources and more rounds of repeated CTTIs are needed to reach the Zero-COVID status. If the CTTI strategy is implemented 11 days after the virus is introduced, compared with the 8-day scenario, the cumulative number of infections can increase about 10 times (from 3585 to 39,686), and the number of individuals needed to be isolated will increase about 8 times (from 40,865 to 367,322). Although the detection is only delayed for 3 days, 16 rounds of citywide testing are needed instead of 10 rounds, even under the strictest implementation of the CTTI policy. Early detection to identify the local epidemics will significantly reduce the health burden of the society for a faster return to normal life.

On the other hand, the considerable effect of the CTTI strategy is also possible in regions where a large outbreak is ongoing (the following case of Jilin). It still may successfully curb the transmission, but with more resources. In fact, this strategy can mitigate and reduce the wave trend by isolating not just cases, but also their contacts and, most importantly, asymptomatic cases, which run the risk of being undetected if a strict testing procedure is not realized.

### The outcome of partially implemented CTTI

**Fig. 4:**
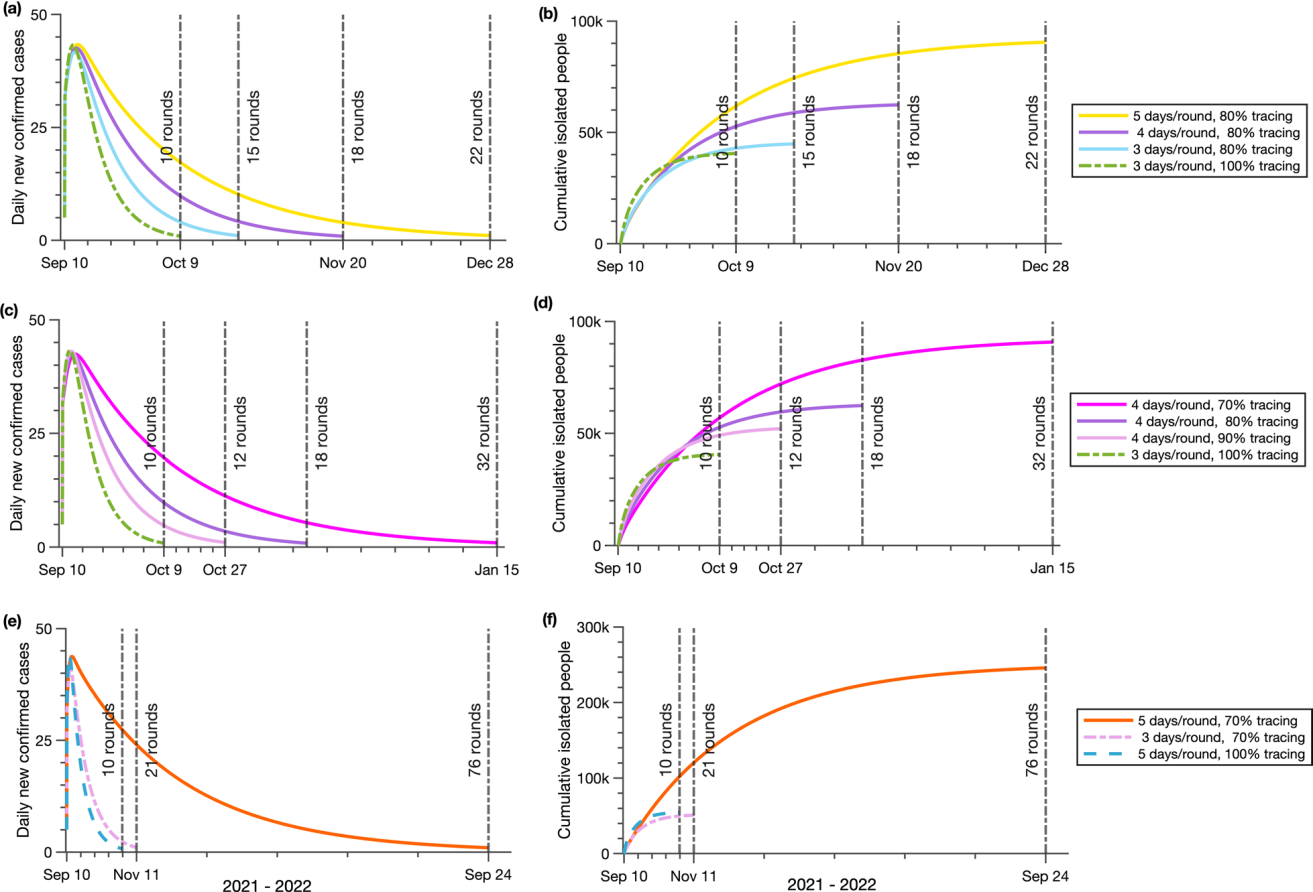
The effect of efficiency of testing and contact tracing on the containment of the COVID-19 epidemic. The daily new confirmed cases (**a**, **c,** **e**), the cumulative isolated people (**b**, **d**, **f**) and the rounds of citywide testing are reported under different efficiency of testing and contact tracing

We explore the effect of test capacity and contact tracing, heavily relying on the public health resources, on eliminating transmission under the assumption of 8 days free transmission stage (Fig. 4). We observe that the testing capacity is crucial for the implementation of the citywide testing and Zero-COVID strategy. The virus in the local area can be quickly eradicated in 30 days under the scenario with perfect CTTI. In a more realistic case with 80% of contacts traced, if one citywide testing is completed in 5 days, it takes 22 rounds of citywide testing and 110 days to eradicate the virus. Compared to a 3-day scenario, the total number of infections will increase by 122% (from 6246 to 13,846), and the total number of isolated individuals will increase by 101% (from 45,466 to 91,313) (Fig. 4a, b). With limited testing capacity and a long period of completing a round of testing, we cannot detect the asymptomatic and presymptomatic cases fast enough, and the exposed individuals will develop infectiousness during the testing round, which makes more people infected.

In addition, effective contact tracing will shorten the time needed to eliminate the local epidemic. If one round of citywide test is finished in 4 days (Fig. 4c, d) and 90% of the primary and secondary contact of confirmed cases is traced, then the local transmission is controlled in 48 days after 12 rounds of testing, with 6662 total infections. However, if the efficiency of contact tracing is reduced to 70%, then the number of rounds of citywide testing needs to be significantly increased to 32 to eliminate the local infections, and the total infections and the number of total isolated individuals will increase by 136% and 75% respectively.

With a limited public health budget, the allocation of resources is paramount. We compare two strategies: test targeted (complete one round citywide test in 3 days, but only trace 70% contacts) and contact tracing targeted (trace 100% contacts, but complete one round citywide test in 5 days). We find that the tracing targeted strategy is more efficient in containing the local outbreak. The tracing targeted strategy ends the epidemic 13 days earlier with fewer infections (30% decrease), though more isolated people (6% increase, Fig. 4e, f).

Nevertheless, substantial resources are needed to achieve zero COVID community transmission if the CTTI is not well enforced (Fig. 4e, f). When the public health resources are seriously limited, the time for one round of citywide test is 5 days, and only 70% of contacts can be traced, then we will need 76 rounds of citywide testing and 380 days to end the local epidemic. The total number of quarantined people will reach 246,363. In this case, it is impossible to eliminate all the cases in the local area, taking the discontent of people towards repeated testing into consideration, and the cost of containment is enormous. However, it is also crucial to test people as much as possible (the partial enforcement of the CTTI strategy), contributing to the epidemic mitigation, although not elimination.

### Challenge of containing the more aggressive Omicron variant of COVID: A case in Jilin Province, China

**Fig. 5:**
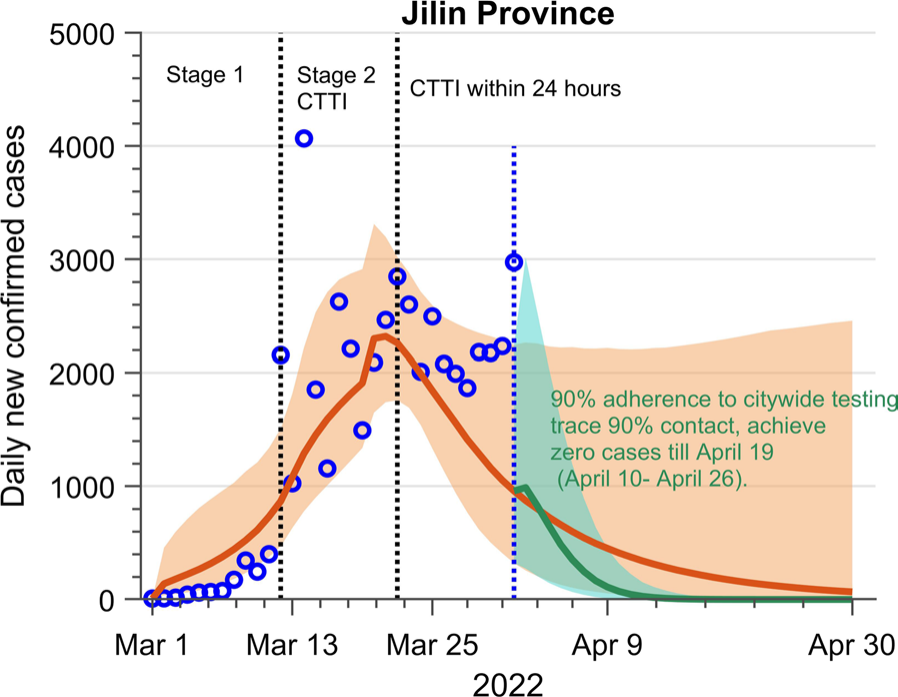
The COVID-19 daily incidence over time in Jilin province, China. The blue circle represents observed data (confirmed + asymptomatic, from March 1 to April 1, 2022). The solid orange line and shaded area represent the median and 95% confidence interval of the model simulations from March 1 to April 30, 2022, based on the parameter estimated from data fitting. The solid green line and shaded area represent the median and 95% confidence interval of the scenario of a 90% adherence rate to citywide testing in Jilin and tracing 90% primary contact and secondary contact in 24 h. The black dot lines separate the different stages considering the change of control measurements. Stage 1: Early stage of the epidemic; Stage 2: control stage with CTTI (strict control measures, completion of citywide testing and contact tracing in 48 h). The blue dot line separates the model validation and projections

We apply our model to simulate the most recent Omicron outbreak in March 2022 in Jilin Province, China, which illustrates the applicability of our model. Figure 5 presents our projection of the Jilin outbreak till April 30, 2022, under the scenarios with the estimated parameter from data fitting and the escalated CTTI strategy. Our results predict that the Jilin outbreak is challenging to achieve zero COVID cases before the end of April under the estimated adherence rate to citywide testing and tracing efficiency, although the citywide testing has been sped up to 24 h (Fig. 5, orange line and shaded area). However, it is possible to eradicate the virus on April 19 (95% *CI*: April 10–April 26) if the compliance rate of citywide testing in Jilin increase to 90% and the 90% of close contacts can be traced and isolated in 24 h (Fig. 5, green line and shaded area). Our results indicate that the CTTI strategy can contain the transmission of the more contagious Omicron variant, but stringent measures (fast and efficient) and continuous efforts are needed.

### Sensitivity analysis

**Fig. 6:**
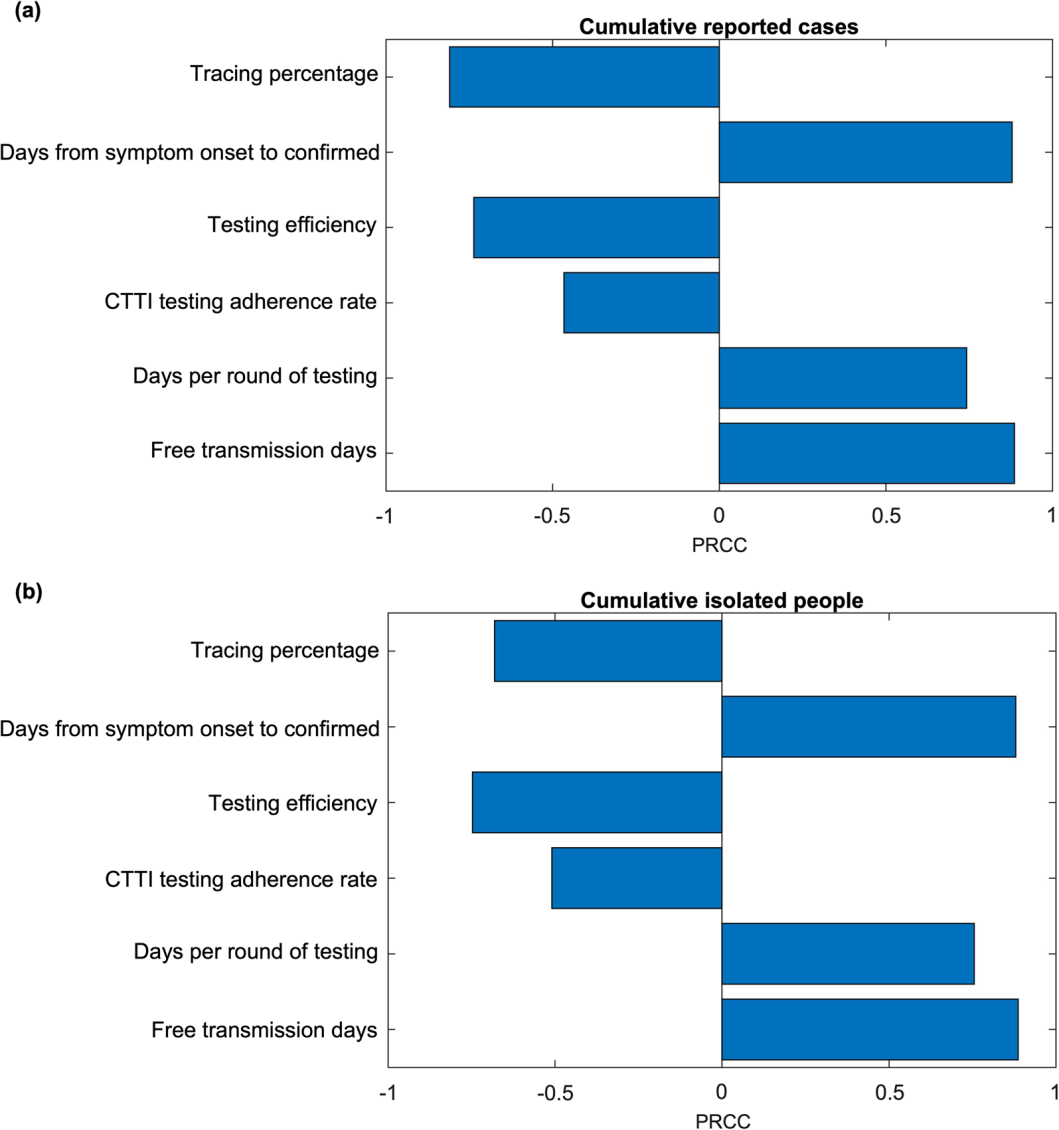
The sensitivity analysis on cumulative reported cases (**a**) and cumulative isolated people (**b**) with different CTTI parameters

Figure 6 shows the Partial Rank Correlation Coefficient (PRCC) of the model parameters, related to the Zero-COVID policy, on the cumulative reported cases (Fig. 6a) and cumulative isolated individuals (Fig. 6b). We observe that the free transmission period presents a positive correlation with the cumulative cases, and the tracing percentage is negatively correlated to the cumulative cases, which also confirms our findings of the importance of early detection and tracing in Figs. 3 and 4, respectively. The days per round of testing are positively correlated to the cumulative isolated people, which illustrates that the testing capacity is paramount. Otherwise, the resources needed to isolate the possible infections will increase substantially.

On the other hand, citywide testing adherence rates are negatively associated with cumulative cases. Also, the test efficiency is negatively associated with the cumulative isolated individuals. A higher test efficiency contributes to the detection of asymptomatic and presymptomatic cases, hence reducing possible community transmission, and finally, less effort of the public health department to isolate the possible infections. The sensitivity analysis illustrates that the zero COVID tolerance policy is challenging, requiring not only stringent public health control measures but also sufficient test capacity, efficient contact tracing, and population adherence to the strategy.

## Discussion

Using a deterministic compartmental model considering the implementation of the Zero-COVID policy in China, defined as the CTTI, we investigate the impact of this strict policy on outbreaks, including cases, severe infections, as well as the number of citywide testing rounds to eliminate the virus. Our calibration results are aligned with the actual situation. In particular, we estimate that the number of rounds needed to reach the containment of the current epidemic in Fujian, China, will be around ten. We find that the CTTI policy, supported by adequate public health resources and population compliance, is efficient in curbing local transmission (Delta and Omicron variants) and, if applied promptly, is able to end an outbreak in a short time, also for the contagious Omicron variant. But this comes with high social and economic costs, which constrain its applicability to other regions. In fact, the effectiveness of the CTTI strategy is further enhanced when combined with timely early case detection. However, the rapid implementation of citywide testing is not the unique measure needed to reduce the local transmission. Efficient contact tracing and isolation of primary and secondary contacts of confirmed cases are also essential to cut off the local transmission. Moreover, cities or regions with limited resources may prioritize the allocation of resources to contact tracing to help control the epidemic.

The CTTI strategy can efficiently eliminate the local transmission, which does not rely on the vaccine availability, the state of the epidemic, and the progression of the disease. This aggressive control strategy breaks the local transmission chain by isolating all the possible infection resources through citywide testing and contact tracing, hence, it can bring any infectious disease epidemic to an end mathematically. The decline in the number of daily new isolated people through testing and tracing indicates that the epidemic is gradually being controlled. Without perfect vaccines, the CTTI policy is the optional way of controlling the outbreak and a possible eradication strategy. However, the public health resources and administration capacity used in eradicating the virus were tremendous. More than 40,000 individuals were isolated, and almost 90 million tests were conducted in the containment of Fujian outbreak in September 2021 [31]. The social and health burden for an optimal strategy to eliminate the infections, including the cost to clear the isolated individuals, calls for further investigations. Moreover, the isolation of confirmed cases should be respected well. Otherwise, the efforts of testing have few benefits without the adherence to isolation, which is also pointed out in Davis et al. (2021) [9]. The isolation of possible infections is the key to the containment of any infectious disease. The elimination of COVID-19 cases requires the effort of the public health department and the compliance of the population to the elimination strategy. Our sensitivity analysis also confirms the importance of compliance of the population for the containment of the epidemic. The CTTI strategy entails mobilizing the whole community, and the supports from both citizens and the public health sectors are indispensable [14, 38]. 

The enforcement of the CTTI strategy is hesitated by authorities due to the high daily testing volume. However, there is the argument that mass testing is feasible even in low-resource rural settings if it meets the requirements of minimal equipment and training [14]. In countries with insufficient resources, implementing a strict CTTI strategy like in China can be almost impossible. However, our work shows that even with less rigorous measures, for example, a limited round of mass testing, the local transmission can be mitigated quickly. And the promotion of self-screening may also be an optional strategy to save resources for testing [38]. In addition, a more effective mass testing strategy can be implemented in the city or region with the constraint of the testing capacity, such as combining with the health code system, prioritizing testing the individuals in higher-risk areas and yellow code holders (Additional file 1: Fig. S1). More accurate testing will further improve the efficiency of containment, thereby reducing people’s suffering and saving medical costs. We also showed that the CTTI strategy is efficient in geographical areas where an ongoing outbreak with high daily new confirmed cases. This strategy can quickly mitigate its epidemic to a certain level, under which less strict measures might be applied. It helps alleviate the pressure on the medical health system, although a resurgence is always visible if cases are not dropped to zero.

The countries that opted for mitigation strategies, like Europe, which is the epicenter of COVID pandemic, are facing a worrying resurgence in COVID cases [42]. Singapore was one of the countries employing the elimination strategy, with aggressive testing and tracing, which helped in bringing the epidemic well under control [43]. However, currently, after lifting restrictions and abandoning the Zero-COVID policy in August 2021 [44], the country is confronted with the largest outbreak since the pandemic has started [45], although it has one of the most successful vaccination rollouts in the world. New Zealand, with a 51% vaccination coverage rate, was also facing a resurgence with Delta variant and decided to live with the virus in October 2021 [46]. Those examples confirm our finding that if a strict Zero-COVID policy is not enforced, there will be multiple outbreaks resulting in more infections and deaths, as well as putting a huge burden on social and health resources.

However, the applicability of the Zero-COVID strategy is doubted, and it is criticized for overreacting to the epidemic and the substantial social and economic cost. It is argued that it is worthwhile to test millions of people to detect several cases. Especially in the later controlled stage, keeping tests seems to have no benefit to the containment of the epidemic. The discontent of populations may be increased along with the repeated testing, and the compliance rate of testing and isolation will be affected by the repeated outbreaks. Hence, the CTTI strategy is an efficient way to control the epidemic, but the optimal way of containment may still need further investigations. On the other hand, the prolonged prevalence of the epidemic may require considerable public health resources and result in immense economic loss. It is difficult to compare the cost between elimination with extensive resources in a short period and mitigation with relatively fewer resources but a more extended prevalence. Further studies in terms of economic assessment may shed some light on this comparison.

Moreover, the high coverage rate of vaccination should be a vital step in the exit strategy. The mitigation of the epidemic is seen with the increase in vaccine coverage. The transmission risk decreases with the vaccination rollouts, although it is challenged by the keep emerging new variants, such as the Delta and Omicron variants [47, 48]. But the relaxation of NPIs is inevitable. People are eager to return to normal life and have begun to accept that we will live with the virus for a long time. The possible resurgence may occur in the circumstance of relaxation of public health control measures and may exacerbate due to the waning immunity of the vaccine. This is the situation that Europe countries were facing [42]. Hence, personal protection, public health control measures, and vaccine booster campaigns must be strictly respected. The combination of the vaccination rollout and the implementation of a partial CTTI strategy when facing the outbreaks could be a more assured exit strategy.

Our analysis has several limitations. Firstly, we do not consider the different testing strategies in the varying levels of risk zones. In the later stage of elimination, the citywide testing has low performance with high cost. Then, only the people in the high-risk zone are tested according to the actual implementation. Our model may overestimate the rounds of CTTI needed due to the spatial heterogeneity. One way is to use individual based modelling or use meta-population models, however this type of spatial modelling would be too complex since we need to define the contact transmission matrix to take care of different population groups and spatial variations in the region considered. We believe that the broad applied mathematics community will continue to seek solutions to use spatial modelling with certain level of heterogeneity to fit the data. We will keep it as our future work too. One other limitation is the vaccination not considered. All the individuals need to do the testing regardless of the vaccination status. The reduced transmission risk is included in its estimation process. Nevertheless, the neglect of the vaccination may also overestimate the resources needed to eliminate the epidemic. In addition, the overlap between the primary contacts and the secondary contact is not considered in the model. The primary contacts of confirmed cases may have contact with each other, and then the number of traced secondary contacts will be reduced. However, it is not an easy task to determine the actual number of primary and secondary contacts, hence we are assuming the average contacts of each person. This simplification does not affect our contact tracing process.

## Conclusions

In summary, our results highlight the importance and efficiency of the CTTI Zero-COVID policy for the containment of the COVID-19 epidemic, by which even the current more aggressive Omicron outbreaks can be contained. Utilizing sufficient resources and early detection of the epidemic, the rapid implementation of the Zero-COVID policy with effective contact tracing, isolation of primary and secondary contacts, the CTTI policy can ensure the elimination of COVID-19 in a short period. But the applicability of CTTI policy to regions with limited resources is constrained, although its partial implementation can help alleviate the epidemic. However, if the outbreak is not wholly eliminated, the resurgence may be experienced in the local area. The combination of CTTI and vaccine may be the feasible exit strategy. Our results also highlight the urgent need for efficient tests, comprehensive contact tracing, and isolation compliance to support the mitigation of global pandemics of COVID.

## Supplementary Information

**Additional file 1:** The supporting materials of implementation of contact tracing in China, the estimated parameters in Fujian case, the initial values of state variables and parameters used in Jilin case and the sensitivity analysis on the number of daily cases and isolated individuals.

## Data Availability

The data used for this study are published by the Fujian Province Health Commission and Government of Jilin Province, publicly available at the following links: http://wjw.fujian.gov.cn/ztzl/gzbufk/yqtb/ and http://www.jl.gov.cn/szfzt/jlzxd/yqtb/index.html.

## Abbreviations

CTTI: Citywide test-trace-isolate
PRCC: Partial Rank Correlation Coefficient
*CI*: Confidence interval
VOC: Variants of concern
NPIs: Non-pharmaceutical interventions
NMSE: Normalized mean square error
LHS: Latin Hypercube Sampling
