# Supplementary material for: Assessing the mechanism of citywide test-trace-isolate Zero-COVID policy and exit strategy of COVID-19 pandemic
Source: Infect Dis Poverty. 2022 Oct 4;11:104. doi: 10.1186/s40249-022-01030-7 (PMC9529335; doi:10.1186/s40249-022-01030-7)
Supplement: Supplementary file 1 — Additional file 1: The supporting materials of implementation of contact tracing in China, the estimated parameters in Fujian case, the initial values of state variables and parameters used in Jilin case and the sensitivity analysis on the number of daily cases and isolated individuals. [file 40249_2022_1030_MOESM1_ESM.pdf]

Assessing the mechanism of citywide test-trace-isolate Zero-COVID policy and exit strategy of COVID-19 pandemic

Pei Yuan[1,2,*],Yi Tan[1,2,*], Liu Yang[1,2,3*], Elena Aruffo[1,2,*], Nicholas H. Ogden[4,2], Guojing Yang[5†], Haixia Lu[6], Zhigui Lin[7], Weichuan Lin[8], Wenjun Ma[9,10], Meng Fan[3], Kaifa Wang[11], Jianhe Shen[8], Tianmu Chen[12], Huaiping Zhu[1,2 †]

Affiliations:

[1] Laboratory of Mathematical Parallel Systems (LAMPS), Department of Mathematics and Statistics, York University, Toronto, Canada
[2] Canadian Centre for Diseases Modeling (CCDM), York University, Toronto, Canada
[3] School of Mathematics and Statistics, Northeast Normal University, Changchun, Jilin, China
[4] Public Health Risk Sciences Division, National Microbiology Laboratory, Public Health Agency of Canada
[5] Key Laboratory of Tropical Translational Medicine of Ministry of Education and School of Tropical Medicine and Laboratory Medicine, the First Affiliated Hospital of Hainan Medical University, Hainan Medical University, Haikou, Hainan, China
[6] School of Arts and Science, Suqian University, Suqian, Jiangsu, China
[7] School of Mathematical Science, Yangzhou University, Yangzhou, Jiangsu, China
[8] School of Mathematics and Statistics, Fujian Normal University, Fuzhou, Fujian, China
[9] Department of Public Health and Preventive Medicine, School of Medicine, Jinan University, Guangzhou, Guangdong, China
[10] Disease Control and Prevention Institute, Jinan University, Guangzhou, Guangdong, China
[11] School of Mathematics and Statistics, Southwest University, Chongqing, China
[12] School of Public Health and State Key Laboratory of Molecular Vaccinology and Molecular Diagnostics, Xiamen University, Xiamen, Fujian, China.

∗ These authors contributed equally to this work.

† Corresponding Author: huaiping@yorku.ca, guojingyang@hotmail.com.

4700 Keele Street, Toronto, Ontario, Canada, M3J1P3

Supplementary materials

Health QR code system and contact tracing in China

In February 2020, China rolled out its "health code" app nationwide. The health QR code system with red, yellow, and green color (**Figure 2**), developed by Alibaba and Tencent, efficiently identifies people exposed to COVID-19.Users can access the app through Alipay or WeChat and obtain their health codes. The color-based code can determine people's exposure risks and freedom of movement based on factors like travel history, duration of time spent in the risky zone, and relationships to potential carriers.

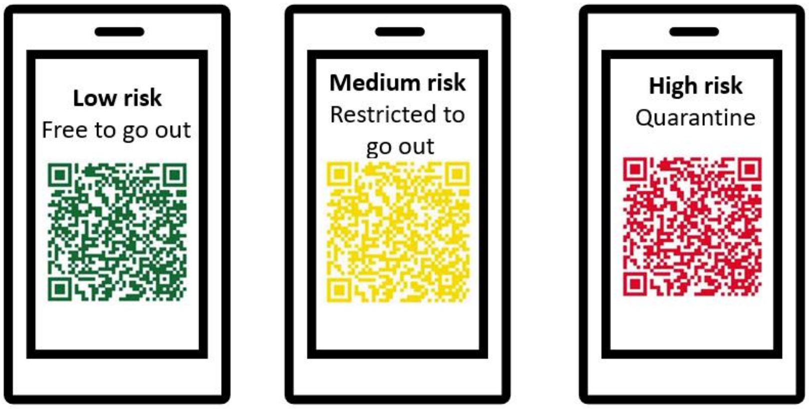

Figure S1 The example of the health QR code system for contact tracing.

The local region is classified into low, medium, and high-risk zone based on the daily new cases, 0, <10 or 1 cluster outbreaks, and >10 or ⩾2 cluster outbreaks in the community within 14 days, respectively [38]. And the cluster outbreaks refer to the occurrence of more than 5 cases in a small area within 14 days [38]. Only the individuals who are in the low-risk zone and have no contact with confirmed cases are assigned with green QR codes and have unhindered access to public spaces. The confirmed cases and their primary (individuals who have close contacts with the confirmed case within 2 days before the onset of symptoms or collecting samples) and secondary contacts (individuals who have close contact with primary contacts within 2 days before the onset of symptoms or collecting samples), and the individuals who stay more than one hour in a high-risk zone are with red QR codes, are required to isolate. People with yellow QR codes are restricted to go out if they stay more than one hour in the medium risk zone or the general contacts (individuals who have been in contact with the index cases in the same public area with a distance greater than 1 meter) [38]. Public places are not allowed to enter by individuals with yellow QR codes. Moreover, the status of the health QR code is updated with time according to the smartphone GPS data.

Suplementary Tables

Table S1: Table of estimated parameters and prior distribution in Fujian

Parameters	Prior range	Distribution	References
Probability of transmission per contact (β)	$0.06 - 0.12$	Uniform	Assumed
The number of contacts per individual per day in the first stage (c_1)	$10 - 20$	Uniform	Assumed
The number of contacts per individual per day in the second stage (c_2)	$5 - 15$	Uniform	Assumed

Table S2: Model variables and parameters for Jilin

Notation	Description	Value	Sources
**Variables and their initial values**			
$S(t)$	The number of susceptible individuals at day t	24073452	Data[1]
$E(t)$	The number of exposed individuals at day t	1	Assumed
$A(t)$	The number of asymptomatic infectious at day t	0	Assumed
$I_1(t)$	The number of presymptomatic infectious individuals at day t	0	Assumed
$I_2(t)$	The number of symptomatic infectious individuals at day t	0	Assumed
$I_d(t)$	The number of confirmed and isolated individuals at day t	0	Assumed
$R(t)$	The number of recovered individuals at day t	0	Assumed
$I_d(T_1)$	The number of confirmed and isolated individuals at day T_1	8	Data[2]
**Parameters for COVID-19 in Jilin**			
τ_1	Average time spent in the exposed state, E, days, for Omicron variant	3-1.5	Ref[3,4]
τ_2	Average time spent in the presymptomatic infected state I_1, days, for Omicron variant	1.5	Ref[5]
a	Proportion of infected people who will develop symptoms	0.6	Data[2]

b	Infectiousness of asymptomatic individuals compared to symptomatic infections	0.75	Ref[6]
η	The testing efficiency towards asymptomatic infections A and presymptomatic infections I_1	0.67	Ref[7]
$\xi_1 p_H$	Proportion of severe infections	0.001	Data[2]
$\xi_2 p_D$	Proportion of confirmed patients in ICU died	0.00008	Data[2]
u_1	Days for developing severe symptoms and need ICU care after diagnosed	8	Ref[8]
u_2	Length of stay for severe patients in ICU before died	10 days	Ref[9]
u_{I_2}	The average days from symptom onset to diagnosis in Jilin	1 days	Assume
γ_A	Recovery rate of asymptomatic infectious individuals	Stage 1: 0<br>Stage 2: 1/6.5	Value of γ and τ_2
γ	Recovery rate of symptomatic infectious individuals	1/5	Ref[10]
γ_d	Removed rate of non-ICU (hospitalized) patients	1/5	Ref[10]
γ_H	Recovery rate of ICU (hospitalized) patients	1/7	Ref[11]
α_2	The adherence rate of symptom-driven testing in Jilin	Stage 2-3: 1	Assumed
P	Total number of populations in Jilin province	24073453	Data[1] **Error! Reference source not found.**
T_0	Time when the virus imported into epidemic areas in Jilin province	Feb. 20, 2022	Assumed
T_1	Time when the first confirmed Omicron cases were detected in Jilin province	Mar. 1, 2022	Data[2]
T_Q	The period of isolation for those traced individuals	14	Ref[12]
T	The time needed to complete one round of citywide test	Stage 1-2: 2<br>Stage 3: 1	Ref[13]
**Estimated parameters (Median, 95% CI)**			
β	Probability of transmission per contact	0.1163 (95% CI: [0.0920, 0.1476])	
c	The number of contacts per individual per day in each stage	Stage 1: 12.3084 (10.0894, 14.8163)<br>Stage 2-3: 8.7575 (5.3450, 11.8226)	

α_1	The adherence rate of citywide testing in Jilin	0.4029 (0.15, 0.6794)
α_2	The adherence rate of symptom-driven testing in Jilin	Stage 1: 0.4641 (0.1457, 0.6898)
p_c	The percentage of contact tracing in Jilin	Stage 1: 0.3296 (0.1082, 0.6966)<br>Stage 2-3: 0.7775 (0.5068, 0.9864)

Table S3: Table of estimated parameters and prior distribution in Jilin

**Parameters**	**Prior range**	**Distribution**	References
Probability of transmission per contact (β)	$0.09 - 0.15$	Uniform	Assumed
The number of contacts per individual per day in the first stage (c_1)	$10 - 15$	Uniform	Assumed
The number of contacts per individual per day in the second and third stage (c_2)	$5 - 12$	Uniform	Assumed
The adherence rate of citywide testing in Jilin in the second and third stage (α_1)	$0.1 - 0.7$	Uniform	Assumed
The adherence rate of symptom-driven testing in Jilin in the first stage (α_2)	$0.1 - 0.7$	Uniform	Assumed
The percentage of contact tracing in Jilin in the first stage (p_c)	$0.1 - 1$	Uniform	Assumed
The percentage of contact tracing in Jilin in the second and third stage (p_c)	$0.1 - 1$	Uniform	Assumed

Sensitivity analysis

Due to daily cases being time series, it's hard to conduct sensitivity for the whole chain. We select four different times ($t = 10, 20, 30, 40$) to reflect the sensitivity for daily cases. We observe that on day 10, tracing percentage is positively correlated to the isolated cases. This is reasonable as there will be more people traced and isolated with a high tracing percentage in the early days of implementing CTTI.

Table S4. PRCC analysis for cumulative and daily cases

Outcome variabels / PRCC / Input parameters	Cumulative cases	Infected cases at day t			
		10	20	30	40
Tracing percentage	-0.7992	-0.6424	-0.7427	-0.7407	-0.8074
Days from symptom onset to confirmed	0.8747	0.8583	0.8511	0.8343	0.8809
Testing efficiency	-0.7309	-0.1885	-0.6360	-0.6632	-0.7490
CTTI testing adherence rate	-0.5014	-0.1737	-0.4293	-0.4380	-0.4916
Days per round of testing	0.7506	0.4697	0.6953	0.7027	0.7669
Free transmission days	0.8879	0.9917	0.9558	0.8943	0.8706

Note: All the parameters are significant at the 0.05 confidence level ($p \leq 0.001$).

Table S5. PRCC analysis for cumulative and daily isolated people at day t

Outcome variabels / PRCC / Input parameters	Cumulative isolated people	Isolated people at day t			
		10	20	30	40
Tracing percentage	-0.6810	0.9220	-0.3636	-0.3552	-0.5466
Days from symptom onset to confirmed	0.8788	0.7436	0.8887	0.8884	0.8710
Testing efficiency	-0.7472	-0.1545	-0.6742	-0.7261	-0.7288
CTTI testing adherence rate	-0.5093	-0.0932	-0.4456	-0.4828	-0.4456
Days per round of testing	0.7549	0.2088	0.7046	0.7412	0.7235
Free transmission days	0.8866	0.9974	0.9924	0.9780	0.9481

Note: All the parameters are significant at the 0.05 confidence level ($p \leq 0.001$).

Reference

1. Statistic bureau of Jilin. Bulletin of the 7th National Census of Jilin Province (No.2). 2021. http://tjj.jl.gov.cn/tjsj/qwfb/202105/t20210524_8079098.html. Accessed 03 Apr 2022.
2. Government of Jilin Provinve. COVID-19 Epidemic situation in Jilin Province. 2022. http://www.jl.gov.cn/szfzt/jlzxd/. Accessed 13 March 2022.
3. Jansen L, Tegomoh B, Lange K, Showalter K, Figliomeni J, Abdalhamid B, et al. Investigation of a SARS-CoV-2-B.1.1.1.529 (Omicron) Variant Cluster—Nebraska-November-December 2021. Morb. Mortal. Wkly. Rep. 2021;70(5152):1782.
4. Brandel LT, MacDonald E, Veneti L, Ravio T, Lange H, Naseer U, et al. Outbreak caused by SARS-CoV-2 Omicron variant in Norway, November to December 2021.Euro Surveill. 2021;26(50):2101147.
5. Lee JJ, Choe YJ, Jeong H, Kim M, Kim S, Yoo H, et al. Importation and transmission of SARS-CoV-2 B1.1.529 (Omicron) variant of concern in Korea, November 2021. J Korean Med Sci. 2021 Dec 27;36(50):e346.
6. Johansson MA, Quandelacy TM, Kada S, Prasad PV, Steele M, Brooks JT, et al. SARS-CoV-2 transmission from people without COVID-19 symptoms. JAMA Netw. Open. 2021;4(1):e2035057-e2035057.
7. Holborow A, Asad H, Porter L, Tidswell P, Johnston C, Blyth I, et al. The clinical sensitivity of a single SARS-CoV-2 upper respiratory tract RT-PCR test for diagnosing COVID-19 using convalescent antibody as a comparator. J. Clin. Med. 2020;20(6):e209.
8. Carvalho-Schneider C, Laurent E, Lemaignen A, Beaufils E, Bourbao-Tournois C, Laribi S, et al. Follow-up of adults with noncritical COVID-19 two months after symptom onset. Clin. Microbiol. Infect. 2021;27(2):258-263.
9. China Business Network. China reported a new COVID-19 death in a patient with serious underlying diseases for the first time in more than a year. 2022. https://www.yicai.com/news/101354151.html. Accessed 03 April 2022.
10. Mint. How long does it take to recover from Omicron? Centre reveals data. 2022. https://www.livemint.com/news/india/how-long-does-it-take-to-recover-from-omicron-centre-reveals-data-11642682192151.html. Accessed 03 April 2022.
11. CBC news. New COVID-19 cases due to Omicron in Ontario expected to peak this month, health minister says. https://www.cbc.ca/news/canada/toronto/covid-19-ontario-jan-19-2022-elliott-moore-update-1.6320029. Accessed February 27, 2022
12. Government of Jilin Province. Notification of COVID-19 Prevention and Control in Jilin Province. 2021. https://www.jl.gov.cn/zw/tzgg/gsgg/gg/202101/t20210124_7923346.html. Accessed 13 March 2022.
13. Government of Jilin City. COVID-19 Epidemic situation in Jilin City. 2022. http://www.jlcity.gov.cn/zt/qtzt/kjxgfy/yqtb/. Accessed 13 March 2022.
